# F132. IDENTIFICATION OF PATIENTS WITH RECENT ONSET PSYCHOSIS IN KWAZULU NATAL, SOUTH AFRICA: A PILOT STUDY WITH TRADITIONAL HEALTH PRACTITIONERS AND DIAGNOSTIC INSTRUMENTS

**DOI:** 10.1093/schbul/sby017.663

**Published:** 2018-04-01

**Authors:** Wim Veling, Jonathan Burns, Hans W Hoek, Ezra Susser

**Affiliations:** 1 University Medical Center Groningen; 2 University of Exeter; 3 University of Groningen; 4 Columbia University

## Abstract

**Background:**

There is considerable variation in epidemiology and clinical course of psychotic disorders across social and geographical contexts. To date, very little data is available of low- and middle-income countries (LMICs). Obtaining valuable evidence from under-represented regions such as Sub-Saharan Africa holds the promise of advancing our knowledge and understanding of psychosis and will provide a strong basis for redressing inequities in service provision for people with psychotic disorders living in LMICs. Many patients in these countries remain undetected and untreated, partly due to lack of formal health care facilities. This study in rural South Africa aimed to investigate if it is possible to identify patients with recent onset psychosis in collaboration with traditional health practitioners (THPs).

**Methods:**

We developed a strategy to engage with THPs. Key to the collaboration between psychiatry, THPs and the local community, was the building of trust by recognizing and acknowledging local authorities, mutual respect for health constructs, taking time to find common ground, and adaptation of the procedures to sociocultural norms. Fifty THPs agreed to collaborate and were asked to refer help-seeking clients with recent onset psychosis to the study. At referral, the THPs rated probability of psychosis (“maybe disturbed” or “disturbed”). A two-step diagnostic procedure was conducted, including the self-report Community Assessment of Psychic Experiences (CAPE) as screening instrument, and a semi-structured interview using the Schedules for Clinical Assessment in Neuropsychiatry (SCAN). Accuracy of THP referrals, and test characteristics of the THP rating and the CAPE were calculated.

**Results:**

In six months, 149 help-seeking clients were referred by THPs, of which 44 (29.5%) received a SCAN DSM-IV diagnosis of psychotic disorder. The positive predictive value of a THP “disturbed” rating was 53.8%. Test characteristics of the CAPE were poor.

**Discussion:**

This pilot study in rural South Africa found that it is possible to identify patients with recent onset psychosis in collaboration with THPs. THPs not only grasped the concept of psychosis, they recognized “being disturbed” as a condition that is often difficult to treat and for which collaboration with psychiatric mental health care might be beneficial. By contrast, the CAPE performed poorly as a screening instrument. Collaboration with THPs is a promising approach to improve detection of patients with psychosis in LMIC.

